# Identification of Two Polysaccharides from *Prunella vulgaris* L. and Evaluation on Their Anti-Lung Adenocarcinoma Activity

**DOI:** 10.3390/molecules15085093

**Published:** 2010-07-27

**Authors:** Liang Feng, Xiao-Bin Jia, Feng Shi, Yan Chen

**Affiliations:** 1 Key Laboratory of Delivery Systems of Chinese Meteria Medica, Jiangsu Provincial Academy of Chinese Medicine, Jiangsu, Nanjing, 210028,China; E-Mails: shifeng_1985_wcl@163.com (F.S.); ychen202@yahoo.com.cn (Y.C.); 2 Biotechnology Labortory of Chinese Medicine, Macau University of Science and Technology, Macau, China; E-Mail: wenmoxiushi@163.com (L.F.)

**Keywords:** * Prunella vulgaris *L., polysaccharide, GC, anti-lung adenocarcinoma, immune function

## Abstract

*Prunella vulgaris* L. (PV) has been used for tumor therapy in Traditional Chinese Medicine for centuries, however, systematic research on extracted PV polysaccharides believed to possess various biological activities, as well as their preventive and anti-tumor effects on lung cancer has not been reported. In this study, two polysaccharides (P31 and P32) were isolated from the aqueous extract of PV and purified through ethanol precipitation, followed by deproteination using DEAE-52 gel-filtration chromatography. The main monosaccharide composition of polysaccharide P32 was analyzed by GC. It was found that polysaccharide P32 consisted of rhamnose, arabinose, xylose, mannose, glucose and galactose in a molar ratio of 3.46:49.32:58.91:0.43:2.64: 3.11, respectively. In order to evaluate polysaccharide P32’s anti-lung adenocarcinoma activities and immunomodulation effects, a *C57BL/6* mouse-Lewis lung carcinoma (LLC) model was established and investigated. Our results showed that polysaccharides of PV had anti-lung cancer activity and could increase the thymus index and the spleen index in tumor-bearing mice, suggesting possible immunomodulation effects.

## 1. Introduction

*Prunella vulgaris* L. (PV) belongs to the Labiatae family of perennial plants and is widely distributed in Asia and Europe [1,2]; its dried spikes are often utilized in Traditional Chinese Medicine. In the Chinese Pharmacopoeia, PV is commonly used to treat headaches, high blood pressure, diseases of the lymphatic system, goiter and tuberculosis. It has long been used clinically in China for the prevention and therapy of non small-cell lung cancer. Currently, PV is considered more compatible with other anti-tumor herbs in lung cancer treatment. It contains components such as terpenoids, flavonoids, polyphenols [3] and polysaccharides that are known to be associated with tumor inhibitory effects. Polysaccharides is some of its main bioactive compounds that possesses immunoregulatory [4,5], anti-inflammatory [6,7], anti-virus [8] and antioxidant [9] activities. In general, qualitative and quantitative analyses of the polysaccharide contents in PV are performed using methods such as colorimetry and high performance liquid chromatography (HPLC). Gas chromatography (GC) is a convenient method also widely employed for the identification of polysaccharide compositions [10,11]. In this study, the compositions of purified polysaccharides extracted from PV were analyzed by GC. Moreover, the effects of these polysaccharides on lung adenocarcinoma and on immune regulation were investigated in vivo using the C57BL/6 mouse-Lewis lung carcinoma (LLC) model.

## 2. Results and Discussion

### 2.1. Isolation of polysaccharide from PV

**Figure 1:**
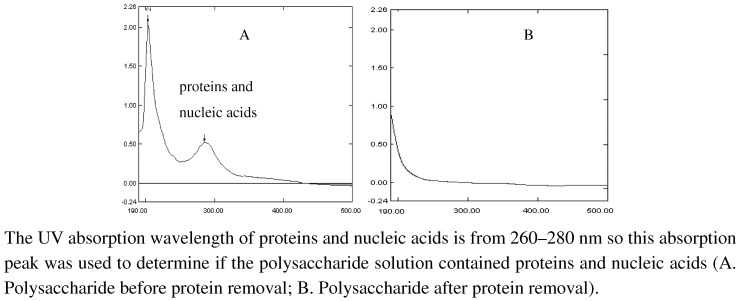
The UV absorbance spectra of polysaccharide before and after deproteinization.

Proteins were removed using the trichloroacetic acid method and the Sevag method (applied once) [12,13]. Samples were scanned at a wavelength of 190–600 nm. As shown in Figure 1, no presence of proteins and nucleic acids were detected after protein removal, as indicated by the disappearance of the absorption at 260–280 nm. 

### 2.2. Molecular weight determination of polysaccharide

**Figure 2:**
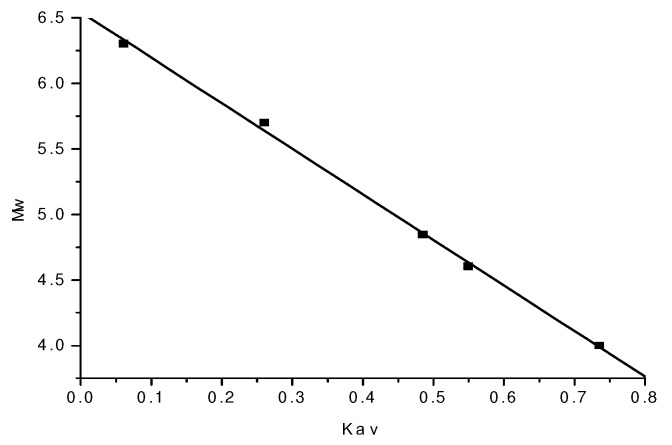
The standard curve of Mw and Kav.

A standard curve was generated based on the Mw, Ve and Kav of dextran standards with various molecular weights (Table 1) as follows: Y = -3.4754X + 6.5431 (r = 0.9991, where Y is the logarithm of molecular weight, and X is the Kav value. The standard curve is shown in Figure 2. 

**Table 1:** The computed value of dextrans,blue dextran and glucose Mw,Ve and Kav.

Dextran	Mw	LgMw	Ve ( min)	Kav=(Ve-Vo)/(Vt-Vo)
T-10	10,000	4	12.643	0.7353
T-40	40,000	4.602	11.344	0.5494
T-70	70,000	4.845	10.895	0.4852
T-500	500,000	5.699	9.325	0.2606
T-2000	2,000,000	6.301	7.930	0.061

Note: Ve was retention time of dextrans; Vo was retention time of blue dextran; Vt was retention time of glucose at 14.495.

**Figure 3:**
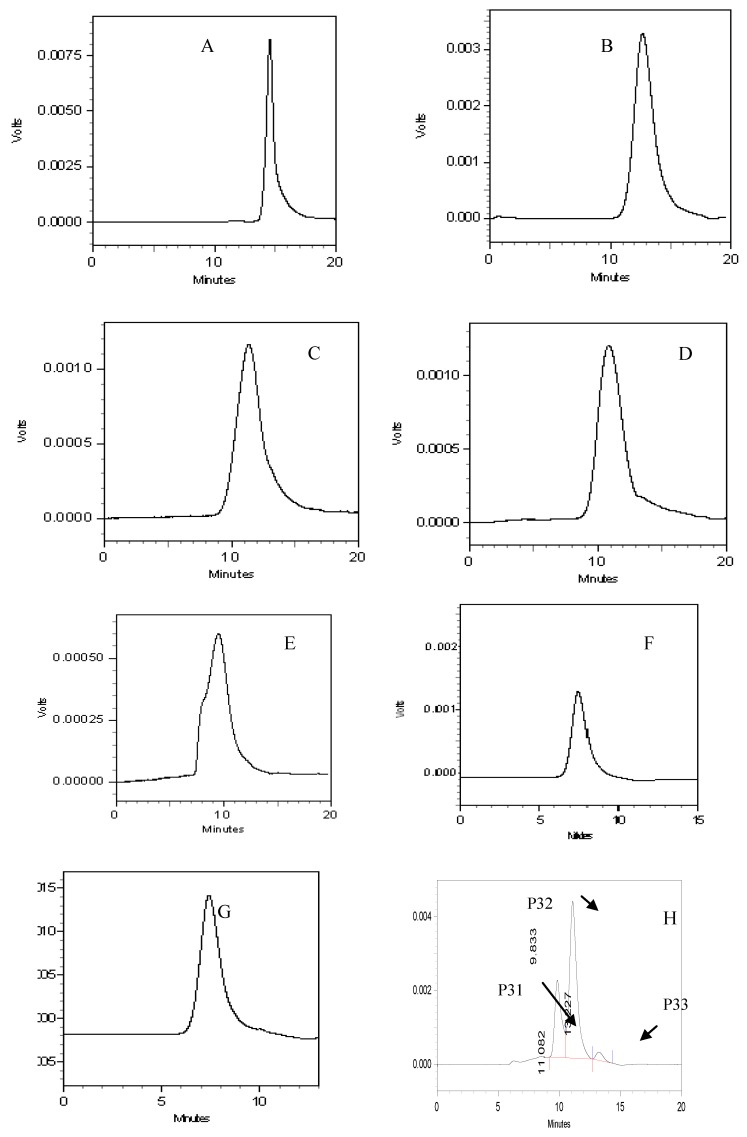
HPLC chromatograms of glucose (A), T-10 (B), T-40 (C), T-70 (D), T-500 (E), T-2000 (F), blue dextran (G), PV (H). T-10, T-40, T-70, T-500 and T-2000 correspond to different molecular weight dextran standards listed in Table 1 with M_w_ = 10,000, 40,000, 70,000, 500,000 and 2,000,000, respectively.

Three polysaccharide components (P31, P32, P33) were observed in the HPLC chromatograms (Figure 3) with molecular weights of 242,641, 58,060 and 4,980, respectively (Table 2).

**Table 2:** The retention time and molecular weight of polysaccharides from PV.

Components	Retention time(min)	Molecular weight
P31	9.833	242,641
P32	11.082	58,060
P33	13.227	4,980

**Figure 4:**
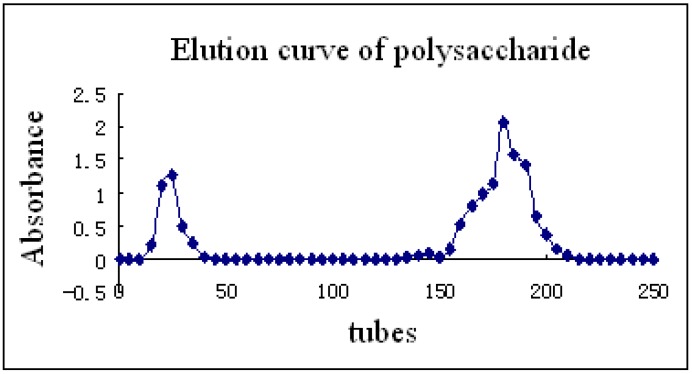
Absorbance by tube number.

In the purification of polysaccharides, DEAE-52 cellulose was used in order to obtain the final polysaccharide fractions (P31, P32). The elution curve (Figure 4) has a symmetrical peak shape, indicating good separation. The results indicate that polysaccharide P32 (between 150 -200 tubes) was the main component; therefore, it was selected for further characterization.

### 2.3. Monosaccharide composition of polysaccharide and its molar ratio

**Figure 5:**
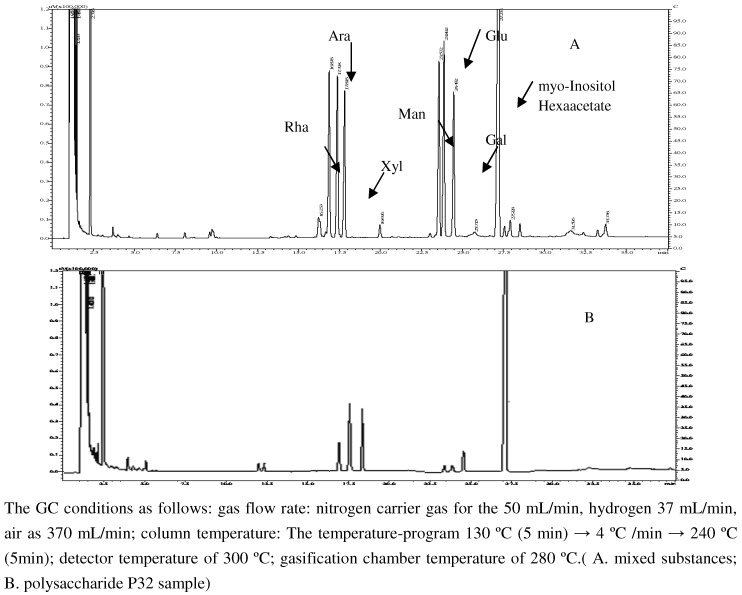
The gas chromatogram of mixed substances and polysaccharide P32 of *PV.*

Polysaccharide P32 was analyzed by GC and the results are shown in Figure 5. Each monosaccharide peak in the order of increasing retention time was identified as rhamnose, arabinose, xylose, mannose, glucose, galactose and myo-inositol hexaacetate. The corresponding retention times were 16.88, 17.38, 17.82, 23.57, 23.89, 24.48 and 27.28 min, respectively. The molar ratio of rhamnose: arabinose: xylose: mannose: glucose: galactose was 3.46: 49.32: 58.91: 0.43: 2.64: 3.11. 

### 2.4. In vivo anti-lung adenocarcinoma activity of PV polysaccharide

**Figure 6:**
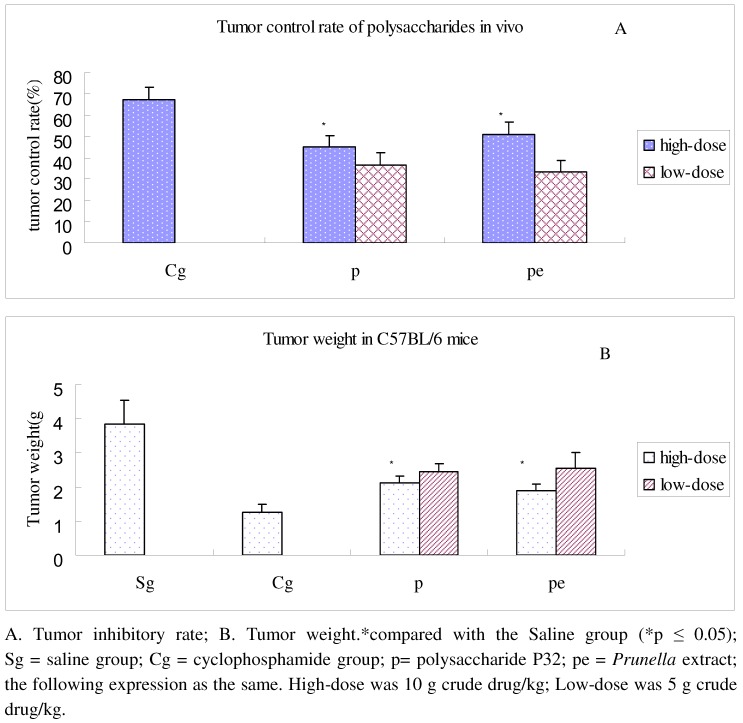
Tumor inhibitory rate of polysaccharide P32 in the C57BL/6 mice.

As can be seen in Figure 6, both the polysaccharide P32 and *Prunella* extract high-dose groups showed significant tumor inhibiting effects in adenocarcinoma tumor-bearing C57BL/6 mice, compared with the saline group (p ≤ 0.05).

As shown in Table 3, the high-dose polysaccharide P32 group significantly enhanced the thymus index (p ≤ 0.01) in tumor-bearing mice as compared to saline group. A similar effect of polysaccharide P32 on spleen index regulation was observed (p≤ 0.01), as shown in Table 4. The spleen index of the high-dose polysaccharide P32 group was considerably higher compared with the low-dose group (p ≤ 0.01) and the cyclophosphamide group (Cg) (p ≤ 0.01). Our results indicate that polysaccharide P32 of PV could enhance the immune response in tumor-bearing mice, which could contribute to the overall anti-tumor effects of the polysaccharides.

**Table 3:** Evaluation of polysaccharide on thymus index in tumor-bearing mice.

Group	Thymus (mg)	Weight (g)	Thymus index (mg/g)
Sg	35.71 ± 7.87	21.43 ± 1.72	1.67 ± 0.28
Cg	30.00 ± 8.16	20.86 ± 2.54	1.43 ± 0.30
10 g crude drug/kg of p	46.25 ± 7.44	19.88 ± 2.36	2.33 ± 0.22**
5 g crude drug/kg of p	38.57 ± 3.78	20.43 ± 1.72	1.89 ± 0.16
10 g crude drug/kg of pe	41.25 ± 6.40	21.63 ± 2.83	1.92 ± 0.26*
5 g crude drug/kg of pe	34.29 ± 7.87	19.43 ± 3.15	1.76 ± 0.25

*compared with the saline group and Cg (*p ≤ 0.05;**p ≤ 0.01;). p= polysaccharide P32; pe = *Prunella* extract.

**Table 4:** Evaluation of polysaccharide on spleen index in tumor-bearing mice.

Group	Spleen(mg)	Weight (g)	Spleen index (mg/g)
Sg	155.71 ± 16.18	21.43 ± 1.72	7.26 ± 0.31
Cg	105.71 ± 16.18	20.86 ± 2.54	5.06 ± 0.25
10 g crude drug/kg of p	192.50 ± 26.05	19.88 ± 2.36	9.71 ± 0.90**
5 g crude drug/kg of p	150.00 ± 24.49	20.43 ± 1.72	7.32 ± 0.80
10 g crude drug/kg of pe	168.75 ± 34.82	21.63 ± 2.83	7.79 ± 1.19
5 g crude drug/kg of pe	155.71 ± 28.78	19.43 ± 3.15	8.00 ± 0.52

*compared with the saline group and Cg (*p ≤ 0.05;**p ≤ 0.01;), p= polysaccharide P32; pe = *Prunella *extract.

The spleen and thymus are two main immune organs that play an important role in anti-tumor activity [15]. In the tumor immune system, CD4+ T cell activation and proliferation are related to NF-kappaB pathways. Choi *et al* [16] demonstrated that the aqueous extract of PV has anti-tumor activity through the suppression of NF-κB activation. We speculate that polysaccharide P32 can down-regulate expression of NF-κB and inhibit the proliferation of tumor cells. 

## 3. Experimental

### 3.1. General

The following instruments were used in this study: Millipore ultrafiltration instrument (XX814V230), DZF-6051 vacuum oven (Shanghai Jing Wang Experimental Equipment Co., Ltd.), Model 6820 gas chromatograph system (Agilent Inc.), RTX-5 Sil MS GC capillary column, TSK-GEL G4000PW (7.5 × 300 mm) gel filtration column (TOSOH Corporation), UV-2450 UV-visible spectrophotometer (Shimadzu Corporation), ALPHA2-4 freeze-drying equipment (CHRIST Companies, Germany). Sodium hydroxide, hydrochloric acid, chloroform, butanol, acetone, diethyl ether, trichloroacetic acid, and ethanol were purchased from Nanjing Chemical Reagent Co., Ltd. DEAE-52 was purchased from Whatman Company. Glucose, rhamnose, xylose, arabinose, galactose, mannose were purchased from Sinopharm Chemical Reagent Co., Ltd. Remaining reagents were AR grade.

### 3.2. Plant material

Dried spikes of *PV* was purchased from Medicinal Corporation of Bozhou City, Anhui Province (batch number 0711128) and authenticated by Professor D.K. Wu from the College of Pharmacy, Nanjing University of Traditional Chinese Medicine.

### 3.3. Extraction of crude polysaccharides

Dried PV spikes (200.0 g) were accurately weighed and then extracted twice with distilled water (2.0 L) under reflux for 2 h. The extracts were combined and centrifuged (4,000 rpm, 30 min). To the precipitate was added a solution of 85% ethanol (v/v) and the mixture was left standing 12 h and then filtered. The precipitate was washed successively with 100 mL each of 95% ethanol, anhydrous ethanol, acetone, diethyl ether (twice), then dried at 40 ºC to obtain the crude polysaccharide P1 (38.7 g).

### 3.4. Deproteination of crude polysaccharides

Trichloroacetic acid method: Crude polysaccharide solution (100 mL) was added slowly into a 15% trichloroacetic acid solution (v/v) chilled in an ice bath and stirred constantly until the solution was without turbidity. The mixture was kept at 4 ºC for 4 h, and then centrifuged to remove the precipitate. The final pH of the solution was adjusted to neutral by addition of 10% NaOH (5.5 mL). The result was liquid 1.

Sevag method: To an aliquot of liquid 1 (100 mL) was added chloroform (20 mL), followed by *n*-butanol (4 mL). The mixture was shaken by hand for 20 minutes and poured into a separatory funnel. The denatured proteins were removed at the junction of the water and solvent layer. The extraction was repeated two times and the extracts were combined to give liquid 2 (P2).

### 3.5. Molecular weight determination

Polysaccharide and Dextran T standards samples were analyzed on a Shimadzu LC-10AVP HPLC system equipped with a TSK-GEL G4000PW column (7.5 × 300 mm) and a Shimadzu RID-10A detector. The mobile phase used consisted of 3.0 mM sodium acetate solution and the flow rate was 1.0 mL/min. The column temperature was maintained at 25 ºC. Polysaccharide solution was filtered through a 0.45 μm membrane before injection and the injection volume was 10 μL. The HPLC profiles of polysaccharide sample solutions are shown in Figure 3. The retention times and molecular weights of the polysaccharide components are summarized in Table 2. The relative molecular mass was determined according to the standard curve. 

### 3.6. Gel column purification of protein-free polysaccharide

Liquid 2 (P2) was pretreated with DEAE-52 chromatography post-column by eluting with distilled water, 0.05, 0.10, 0.15, 0.20, 0.50, 1.00, 1.50, and 2.00 mol/L NaCl solution using a DHL-A constant flow pump at a flow rate of 1 mL/min. An automatic collection device was used to collect the fractions of 6 mL per tube. The phenol-sulfuric acid method was used and the eluate was monitored at 490 nm until there was no detection of sugar. The combined eluates were filtered through a Millipore ultrafiltration system to remove small molecules, and then vacuum concentrated and freeze-dried to obtain crude polysaccharide. Pretreated P2 was dissolved in distilled water (10 mL) and passed through a DEAE-52 gel-filtration column. The sample was initially eluted with phosphate buffer (pH = 7.2) at a flow rate of 6 mL/min for 100 min (tubes 1–100, fractions were collected at 6 mL per tube), followed by a gradient of 10 mM, l.0 mM, 10 mM NaCl solution (tubes 101-300 at 6 mL per tube). The fractions were monitored using the phenol-sulfuric acid method and the data was plotted. Fractions corresponding to the absorbance peaks were combined, concentrated and lyophilized to yield the final polysaccharide fractions P31 and P32 (total yield: 29.6 g). [14]

### 3.7. Preparation of myo-inositol hexaacetate as the internal standard

A mixture of inositol (3.0 g), hydroxylamine hydrochloride (4.5 g), acetic anhydride (45 mL) and pyridine (3 mL) was stirred for 2 h at 90 ºC in a water bath. The reaction mixture was cooled to room temperature and then poured into ice water (50 mL) to precipitate phytic acid ester. The solid was filtered and washed with distilled water (20 mL), then dried in oven at 100 ºC and stored for further use. The final product was analyzed and confirmed by GC as myo-inositol hexaacetate. 

### 3.8. Hydrolysis and derivatization of monosaccharide and polysaccharide

To a test tube was added monosaccharide (5.0 mg), hydroxylamine hydrochloride (5.0 mg), myo-inositol hexaacetate (5.0 mg) and pyridine (0.5 mL). The reaction mixture was heated for 30 min at 90 ºC in a water bath with vibration. After the reaction cooled to room temperature, acetic acid anhydride (0.5 mL) was added and the reaction was heated at 90 ºC for another 30 min to continue the acetylation reaction to obtain the monosaccharide samples. Polysaccharide P32 sample (5.0 mg) was dissolved in sulfuric acid solution (10 mL, 2 mol/L) and transferred to an ampoule bottle, then the bottle was sealed and heated at 105 ºC in the oven for 8 h. After hydrolysis, the solution was derivatized using the above method described for monosaccharide. The derivatized samples were injected directly into the GC and analyzed.

### 3.9. Anti-lung adenocarcinoma activity of polysaccharide in C57BL/6 mice

Male C57BL/6 mice, 6-8 weeks of age and weighing approximately 18–20 g, were obtained from Shanghai SLAC Laboratory Animal Co., Ltd. and were maintained on a standard environmental condition. They were fed with standard diet and water *ad libitum*. Primary tumors were induced by subcutaneous (s.c.) injection of 10^6^ Lewis cells in 200 μL PBS in the right anterior limb. The mice were intragastrically administered with 0.4 mL of PV extracted polysaccharide P32 solution (high dose: 75 mg/mL; low dose: 37.5 mg/mL), which was equivalent to 5–10 g crude drug. The *Prunella* extract group was intragastrically administered aqueous extract of PV (high dose: 10 g crude drug/kg; low dose: 5 g crude drug/kg). The drugs were administered every day for 14 consecutive days. Positive control mice were injected with 0.2 mL/day of cyclophosphamide (20 mg/kg), while blank control mice received 0.4 mL/day of 0.9% sodium chloride. Mice were weighed before sacrifice, and the tumor, thymus and spleen were gently extracted. The tumor inhibition rate, thymus index and spleen index were then calculated. Tissue samples were stored at -20 ºC for future study.

### 3.10. Statistical analysis

All data were expressed as mean ± SD and analyzed by one-way ANOVA with the spss16.0 software. The significant differences within and between groups were investigated.

## 4. Conclusions

Two polysaccharides extracted from PV (P31 and P32) were successfully separated and purified. The main polysaccharide P32 was found to consist of rhamnose, arabinose, xylose, mannose, glucose and galactose. In addition, polysaccharide P32 was found to exhibit anti-lung cancer activity and could increase the thymus index and spleen index in tumor-bearing mice. The immunomodulation effect may be one of the mechanisms contributing to the observed anti-lung adenocarcinoma activity.
